# Predicting Demographic Contraction as an Upstream Signal for Health-System Planning: A Persistence-Corrected Machine-Learning Framework

**DOI:** 10.3390/healthcare14203494

**Published:** 2026-10-17

**Authors:** Da Hee Kim, Hyung Jong Na

**Affiliations:** 1 Department of Medical Management, Chodang University, 380, Muan-ro, Muan-eup, Muan-gun 58530, Jeollanam-do, Republic of Korea; dream4656@cdu.ac.kr; 2 Department of Accounting and Taxation, Semyung University, Jecheon 27136, Chungcheongbuk-do, Republic of Korea

**Keywords:** demographic contraction, health-system planning, health policy, hospital-bed density, machine learning, persistence correction, permutation importance, fertility

## Abstract

Background/Objectives: Demographic contraction changes the scale and geography of healthcare demand while weakening the fiscal and workforce base that sustains services. The general objective was to evaluate a persistence-corrected one-year-ahead demographic forecasting framework and to determine whether it can provide a defensible upstream signal for health-system planning; the primary model forecasts demographic change rather than health-system capacity itself. Methods: The analytic sample retained 672 of 4616 documented country-year observations (14.6%) from 47 countries (2000–2020), representing a coverage-selected panel with sufficiently complete cross-domain data. Requiring consecutive observations produced a 522-row dynamic panel from 45 countries and 167 one-year-ahead forecasts for 2016–2020. DPC-GB learned deviations from the current population-growth rate under expanding-window validation. Analyses compared momentum and cross-domain feature sets, matched gradient-boosting hyperparameters for direct and correction targets, lagged structural covariates, held country groups out of training, and used country-cluster uncertainty estimates. Incident-onset probabilities were examined only as a supplementary exploratory analysis. Results: Among the feature specifications, momentum plus non-health predictors had the lowest observed RMSE (0.20491), followed by momentum only (0.20503), the full DPC-GB model (0.20531), and momentum plus health predictors (0.20680); persistence had RMSE 0.21920. The full model’s observed RMSE was 6.34% lower than persistence, but the country-cluster bootstrap interval for the absolute difference [−0.0038, 0.0366] crossed zero (one-sided *p* = 0.0968), so improved forecasting across countries was not established. With identical gradient-boosting hyperparameters, direct level prediction had RMSE 0.2259 versus 0.2053 for persistence correction, although their country-cluster difference interval included zero. After Holm correction across four exploratory transfer endpoints, evidence remained for fertility forecasting; hospital-bed density was not significant after adjustment (adjusted *p* = 0.072). Only one incident contraction occurred among 136 eligible forecasts, precluding validation of probability calibration, discrimination, or operating thresholds. Conclusions: DPC-GB is a retrospective proof-of-concept for persistence correction in demographic forecasting, with possible downstream relevance to health-system planning. These data do not establish improved point forecasting across countries. Generalizability is constrained by complete-case selection. Momentum plus non-health predictors yielded the lowest observed RMSE, but its small numerical advantage over momentum only does not establish superiority; the three-feature momentum-only specification provides a parsimonious alternative to the full 42-feature model. Incident-risk probabilities remain exploratory and cannot support validated early-warning or threshold claims.

## 1. Introduction

Demographic change is a public-health systems issue because shifts in the size and age composition of populations alter health-care demand, long-term-care needs, labour supply, fiscal capacity and the spatial organization of services. Public-health responses to population ageing emphasize adaptation of health systems rather than treating demographic change as a purely macroeconomic phenomenon [1]. Health-system projection studies likewise show that future funding, hospital-bed capacity and workforce requirements depend jointly on demographic structure and morbidity trajectories [2], while global burden-of-disease and long-run population forecasts explicitly integrate demographic futures to support health investment and planning [3,4].

Population decline creates a related but distinct planning problem. A shrinking population does not imply proportionate reductions in health-care need: contraction commonly coincides with population ageing, geographic concentration of vulnerable groups, workforce out-migration and largely fixed infrastructure costs. Empirical evidence from shrinking cities indicates that population loss is associated with changes in hospital-bed and physician provision and with inequalities in access to health services [5]. Cross-country evidence also points to links among economic conditions, hospital capacity, health spending and older population structures [6], and the macroeconomic literature on ageing shows that the fiscal base itself responds to demographic composition [7]. For public-health authorities, an early-warning estimate that a jurisdiction is likely to cross from positive to negative population growth can therefore be useful even though it is not itself a health outcome.

Long-horizon demography has well-developed stochastic and Bayesian methods for representing uncertainty in fertility, mortality, migration and total population. Stochastic population forecasts demonstrated the value of replacing a single deterministic path with a distribution of future outcomes [8], and Bayesian systems subsequently produced probabilistic population projections for countries and for the United Nations [9,10,11]. Those frameworks remain the appropriate reference for cohort-component projection. The present study addresses a complementary retrospective question: whether routinely collected cross-domain indicators improve one-year-ahead forecasts of annual population growth beyond demographic momentum. A supplementary incident-onset probability analysis illustrates the limitations imposed by sparse events and is not a validated probabilistic forecasting contribution.

Machine learning is increasingly used in healthcare and public-health analysis because it can integrate heterogeneous data, approximate nonlinear response surfaces, and identify complex interactions. Recent research has emphasized that policy-facing machine learning requires temporal validation, transparent interpretation, bias assessment, and reproducibility [12]. Population-forecasting applications have compared boosting, tree ensembles, regression, and time-series approaches [13], while newer systems have combined boosting with optimization or multi-source data [14]. Three problems are especially consequential when such models are used for population-related health-system planning.

First, a flexible learner must be tested against a strong demographic baseline. Annual population-growth rates are highly persistent, so a model can achieve a very high R^2^ while offering almost nothing beyond the naive forecast that next year will resemble this year. Second, forecast users need to know whether corrections are driven by demographic momentum, health-system indicators or other covariates. Third, probabilities intended for planning require evidence about calibration, discrimination and threshold performance. Prediction-model reporting standards emphasize transparent evaluation [15]; with only one incident contraction in the eligible evaluation sample, this study cannot establish those properties.

We examine a Dynamic Persistence-Correction Gradient Boosting model (DPC-GB). Instead of reconstructing the full growth-rate level, the model begins from persistence and learns the expected departure from that anchor. Tree-based methods are suited to nonlinear tabular data [16,17,18,19,20,21], while support-vector regression provides a complementary nonlinear benchmark [22]. Out-of-sample permutation tests quantify predictive reliance, with caution for correlated predictors [23,24]. Residual-based probability diagnostics are confined to exploratory Supplementary Materials and interpreted in light of event sparsity [25,26,27,28].

The general objective is to evaluate whether persistence correction adds information beyond current population-growth momentum and to define the limits of its possible use as an upstream input to health-system planning. We address three main research questions. RQ1: Does DPC-GB improve one-year-ahead population-growth forecasts relative to persistence and conventional direct level-forecast models under rolling-origin validation? RQ2: When the estimator and hyperparameters are held constant, does correction targeting differ materially from direct level targeting? RQ3: Do health and other structural predictors add incremental accuracy beyond demographic momentum, and does the architecture transfer to selected planning-related endpoints? A separate supplementary analysis describes incident-onset probability outputs without treating calibration, discrimination or threshold performance as validated research contributions.

The contribution is deliberately narrow. The study evaluates an explicit persistence-correction target, an identical-estimator comparison with direct level targeting, country-cluster uncertainty, and health-information ablation and secondary endpoints in a reproducible short-horizon design. The 6.34% observed RMSE reduction does not establish general superiority, and the full cross-domain specification is not the lowest-error feature set. The supplementary onset analysis documents why probability validation is not possible with the observed event count.

### Related Work and Study Positioning

Closely related work falls into four groups: probabilistic demographic projection, machine-learning population forecasting, multi-source subnational demographic prediction, and health-system resource projection. These studies overlap with the present study but answer different forecasting questions. Table 1 makes those distinctions explicit.

The novelty is therefore not the use of machine learning for population forecasting itself. It is the joint test of incremental value beyond persistence, the same-estimator comparison of target formulations, the restriction from prevalent negative growth to true incident contraction, and the explicit demonstration that health-domain inputs do not improve the primary one-year point forecast once momentum is available.

## 2. Materials and Methods

### 2.1. Study Design, Reporting and Data Sources

We conducted a retrospective multi-country forecasting study using annual aggregate data from 2000 through 2020. The researcher-assembled analytical file combines indicators attributed in the supplied data dictionary to the World Bank, World Health Organization (WHO), International Monetary Fund (IMF), Organisation for Economic Co-operation and Development (OECD), and V-Dem. The target is annual population growth in percentage points per year. Twenty predictors cover health, economic, social, institutional, and environmental domains. This is a retrospective analysis of the merged values available in the research file, not a real-time vintage forecast: the package does not retain exact public-release dates or revision vintages for every year-t indicator. Accordingly, temporal target separation prevents direct target leakage but does not by itself prove that every structural indicator would have been available in its final revised value on each historical forecast date.

Before modelling, we performed an integrity audit of the retained merged analytical file and of the derived dynamic panel. The 672 × 24 merged file contained 47 countries covering 2000–2020, no duplicate country-year or code-year keys, no missing or non-finite retained values, a one-to-one mapping between Country and Code, and no violations of the expected 0–1 bounds for the proportion/index variables. We then reconciled all 522 dynamic-panel rows against the merged file: every row had consecutive t − 1, t and t + 1 records; PrevYear and TargetYear equalled t − 1 and t + 1; Target and PG_lag2 matched the corresponding population-growth values; and all contemporaneous fields and first differences reproduced the merged data with zero alignment mismatches. These checks establish internal integrity from the retained merged file forward; they do not reconstruct the upstream acquisition and merge process for the original 4616-row source pool.

**Figure 1:**
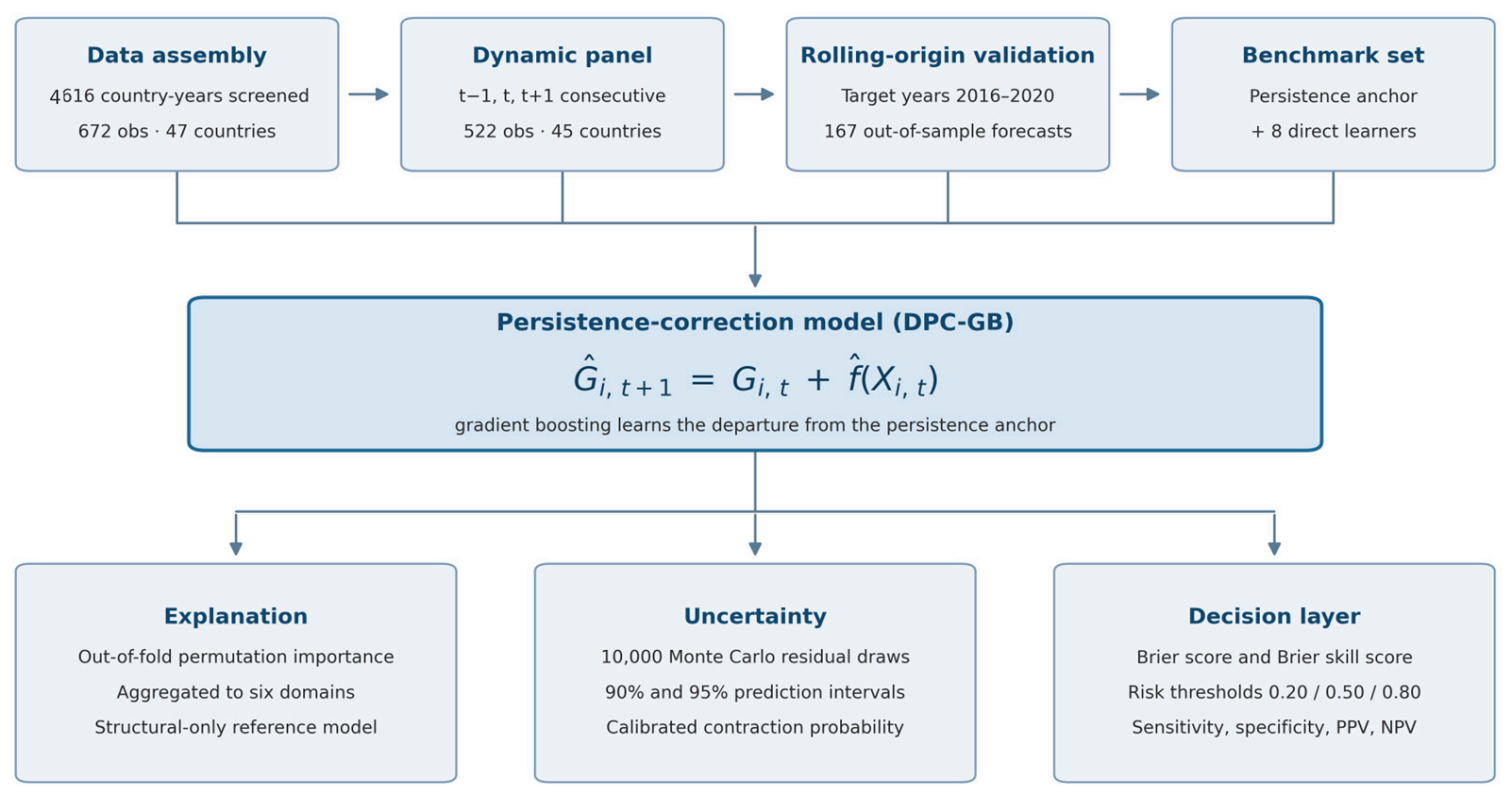
Analytical workflow of the persistence-correction forecasting framework.

The documented source pool contained 4616 country-year observations. Sequential complete-case exclusions for missing health, economic, social, political and environmental information yielded 672 observations from 47 countries. This attrition of approximately 85% is substantial and is treated as a limitation rather than as evidence of global representativeness. The analytical sample should be interpreted as a multi-country panel with sufficiently complete cross-domain data; the included countries and their regional and income composition are listed in Supplementary Table S3. The analytical workflow of the persistence-correction forecasting framework is summarized in Figure 1.

The upper row summarizes data assembly, dynamic-panel construction, temporal validation, and the benchmark set. DPC-GB learns a nonlinear correction to the persistence anchor. The lower layer includes attribution and uncertainty diagnostics; any probability or decision-oriented element is exploratory and reported in the separate Supplementary Materials.

### 2.2. Outcomes and Predictors

The primary outcome was the population growth rate at year t + 1. Health-domain predictors at year t were the total fertility rate, log per capita health expenditure, hospital-bed density (beds per 1000 people) and DPT immunization coverage. Economic predictors were log GDP per capita, share of world GDP, CPI growth and the log GDP deflator. Social predictors were primary and tertiary education expenditure relative to GDP, youth unemployment and total unemployment. Institutional predictors were the V-Dem civil society, equal protection, freedom of expression and access-to-justice indices. Environmental predictors were renewable-energy share, per capita freshwater resources, log greenhouse-gas emissions and log air-pollution exposure. HospitalBeds is a population-normalized hospital-bed-density measure, not an absolute count of beds; it can change mechanically when the population denominator changes.

Secondary planning endpoints were the next-year fertility rate, hospital-bed density (beds per 1000 people), log health expenditure per capita and DPT immunization coverage. These are not presented as direct clinical outcomes. The hospital endpoint is explicitly labelled hospital-bed density rather than “capacity”: density can rise because population falls even if the absolute number of beds does not increase. The present data do not contain absolute bed counts, age- or morbidity-adjusted service need, or utilization; therefore the hospital analysis cannot establish transfer to physical capacity planning.

### 2.3. Dynamic Panel Construction and Leakage Audit

For each country, observations were sorted by calendar year. The forecasting dataset required consecutive observations at t − 1, t and t + 1 so that the information set could include the current level, one lag and recent changes while the outcome remained strictly future. This requirement produced 522 dynamic observations from 45 countries. The outer out-of-sample period comprised target years 2016–2020 and 167 country-year forecasts.

The primary feature set contained current population growth, its lagged level, its first difference, the 20 structural predictors and first differences in the structural predictors. We excluded the first difference in per capita freshwater resources from the main feature set because its correlation with current population growth was approximately −0.992. Because the freshwater measure is expressed per capita, differencing can mechanically encode contemporaneous population change. As an information-availability sensitivity analysis, we additionally replaced the year-t structural levels with their one-year-lagged values while retaining the demographic information available at t. This lagged-covariate analysis reduces reliance on late year-t releases but does not reconstruct true historical data vintages or later revisions.

### 2.4. Persistence Benchmark and the DPC-GB Architecture

The strongest simple benchmark is persistence: the forecast for country i at t + 1 equals its observed growth rate at t. Let G_i,t_ denote annual population growth. The persistence forecast is Ĝ_P_i,t+1_ = G_i,t_. The DPC-GB architecture models only the departure from this anchor. Defining the next-year correction as ΔG_i,t+1_ = G_i,t+1_ − G_i,t_, gradient boosting estimates f(X_i,t_) = E[ΔG_i,t+1_|X_i,t_], and the final forecast is Ĝ_DPC_i,t+1_ = G_i,t_ + $\hat{f}$(X_i,t_).

This formulation narrows the learner’s task. Most short-horizon demographic momentum is absorbed by the benchmark, while machine learning is reserved for nonlinear corrections. DPC-GB used 80 estimators, maximum tree depth 2, learning rate 0.05, Huber loss and seed 20260817. These settings were not selected by searching the 2016–2020 outer evaluation years. They were fixed before evaluation by expert judgement as a deliberately low-capacity, shallow-tree, shrinkage-based configuration for the modest country-year panel, with Huber loss chosen to reduce sensitivity to large residuals. They should be interpreted as a prespecified design choice rather than an empirically optimized configuration.

### 2.5. Benchmark Models

Direct level forecasts were estimated using ordinary least squares, ridge regression, elastic net, support-vector regression with a radial-basis-function kernel, random forest, extremely randomized trees, gradient boosting, and histogram gradient boosting. Their configurations were likewise fixed before the outer evaluation using conventional, moderately regularized benchmark settings; no algorithm-specific hyperparameter search used outer-year performance, and the benchmark ranking should not be read as a definitive comparison of optimally tuned algorithms. Full configurations are reported in Supplementary Table S2. To isolate target formulation from estimator/tuning effects, we additionally fitted a direct gradient-boosting model with exactly the same 80 estimators, depth 2, learning rate 0.05, Huber loss and random seed as DPC-GB; only the prediction target differed.

### 2.6. Temporal Validation and Forecast Metrics

Forecast performance was assessed with expanding-window rolling-origin validation. For target year 2016, the model was trained only on observations whose targets occurred before 2016; the process was repeated sequentially through 2020. No future target year entered the training data for an earlier forecast. This prevents direct target leakage, but it is not equivalent to a vintage-data backtest because final historical values of structural indicators may have been released after the nominal forecast date or revised later. We therefore report a one-year-lagged structural-covariate sensitivity analysis and describe the main evaluation as retrospective temporal validation rather than a fully prospective real-time simulation.

Point accuracy was summarized with mean absolute error (MAE), root mean squared error (RMSE) and R^2^. RMSE improvement relative to persistence was calculated as 100 × [1 − RMSE(model)/RMSE(persistence)]. Absolute and squared-error losses were interpreted as complementary rather than interchangeable because they weight large misses differently [29,30]. We emphasize that R^2^ is a weak diagnostic in this setting: because annual population growth varies far more across countries than within a country across adjacent years, any persistence-anchored forecast attains a high R^2^ almost mechanically. R^2^ is therefore reported for comparability with the existing literature, whereas the improvement relative to persistence is the quantity that carries evidential weight.

### 2.7. Predictive Reliance and Local Attribution Diagnostics

Repeated permutation importance was calculated within each outer test fold for the DPC-GB correction model. Each feature was permuted 30 times, and importance was defined as the resulting deterioration in correction RMSE; fold estimates were weighted by the number of test observations. These values explain model reliance for departures from persistence rather than the full population-growth level. Because correlated variables can exchange importance, no single attribution is interpreted as a unique mechanism. To address country-level interpretability, we also summarized out-of-sample local SHAP values for the correction component, identifying the three largest absolute contributors for each country-year and measuring adjacent-year top-three stability with Jaccard overlap. Local SHAP diagnostics are exploratory and are not presented as causal or fully stable explanations.

A separate structural-only gradient-boosting model was estimated from the 20 level predictors while excluding demographic-history variables. Its predictive accuracy was reported alongside its importance ranking: MAE was 0.3597, RMSE 0.4582 and R^2^ 0.6672 over the 167 outer forecasts, substantially weaker than persistence (RMSE 0.2192). Structural-only importance is therefore interpreted only as the allocation of predictive reliance within a weak model and not as evidence that the leading variable is a strong stand-alone forecasting signal.

### 2.8. Supplementary Exploratory Incident-Onset Analysis

Residual-based incident-onset probabilities were generated only as an exploratory methodological illustration. Eligible forecasts had PopulationGrowth(t) ≥ 0, and onset was defined as PopulationGrowth(t + 1) < 0. Only one incident event occurred among 136 eligible forecasts. This event count cannot validate calibration, discrimination or threshold performance. Monte Carlo construction, probability baselines, ROC and calibration diagnostics, illustrative thresholds, and full-pipeline cluster-refit results are described in the separate Supplementary Materials (Sections S1 and S2, Tables S6 and S8, and Figure S1).

### 2.9. Secondary Planning Endpoints and Health-Information Ablation

The same persistence-correction logic was applied separately to fertility, hospital-bed density (beds per 1000 people), per capita health expenditure, and DPT coverage. Evaluation used the 2016–2020 rolling-origin years and 167 outer observations. The four endpoint analyses were prespecified as exploratory, and one-sided country-cluster bootstrap probabilities were adjusted across the four endpoints with the Holm procedure. A hospital-bed-density result is not interpreted as an effect on, or forecast of, absolute physical capacity.

We also performed a health-information ablation for the primary population-growth outcome: momentum-only, momentum plus health, momentum plus non-health, the full DPC-GB feature set, and a health-only correction. This analysis tests whether health variables increase point accuracy over recent demographic momentum. It is reported in full even where the result is negative, because a public-health framing should not rest on selective performance reporting.

### 2.10. Statistical Uncertainty and Robustness

Country-cluster bootstrap inference used 10,000 resamples of countries for point-forecast RMSE/MAE comparisons and the four secondary endpoints. Additional robustness analyses included matched-hyperparameter direct-versus-correction gradient boosting, one-year-lagged structural covariates, and five-fold country-blocked rolling-origin validation in which held-out countries never appeared in training. The latter assesses within-dataset jurisdiction transfer, not external validation to a new data-generating environment. The separate Supplementary Materials describe the 500 exploratory full-pipeline incident-probability refits and exact threshold intervals; resampling does not supply additional independent incident events.

### 2.11. Reproducibility and Ethics

All verified analyses were implemented in Python 3.12.15 with a fixed seed (20260817). The retained analysis materials are publicly available at https://github.com/Nahj5525/machine-learning-for-early-warning-of-demographic-contraction (accessed on 14 October 2026). The repository snapshot cited for this revision is main-branch commit 3442a32c485209b1f63c1654df59ac33f20598ad (14 October 2026), which contains Reproducibility_Package (Demographic Contraction for Health-System Planning).zip. The package contains the merged analytical data, analytical units, data dictionary, analysis code, outer predictions, tables, figures, environment information and checksums, and supports reproducibility from the retained merged research file forward. The retained files preserve the merged-file identifiers, analytical units/scales and source organizations, but not variable-specific upstream agency series codes, original download dates/source vintages, or the original cross-agency extraction/merge code; unavailable provenance elements are reported as unavailable rather than inferred.

The study used secondary country-level aggregate statistics and did not involve identifiable human participants, clinical interventions or access to individual health records. Human-subject consent and participant-recruitment procedures were therefore not applicable.

## 3. Results

### 3.1. Sample and Analytical Design

The complete-case file contained 672 country-year observations from 47 countries. Requiring consecutive t − 1, t and t + 1 observations produced a 522-observation dynamic panel from 45 countries. The outer evaluation contained 167 forecasts across target years 2016–2020 (Table 2).

Attrition from the documented 4616-row source pool to 672 complete cases was approximately 85%. The retained sample is highly concentrated in settings with stronger statistical infrastructure: 35 of 47 countries (74.5%) are high income, 33 (70.2%) are in Europe and Central Asia, no low-income country is retained, and only one lower-middle-income country is retained. Complete-case coverage also increases over time, from 19 country-year observations in 2000 to 42 in 2017; 231 of 672 observations (34.4%) occur in 2015–2020. Within the retained file, median population growth is 0.575 percentage points/year (IQR 0.081–1.140), 21.4% of observations have negative growth, and median log per capita health expenditure is 7.477 (IQR 6.578–8.352), equivalent to approximately US$1767 (US$719–4241) on the pre-log scale. The original row-level pre-merge file containing the excluded observations was not retained, so excluded-row values cannot be reconstructed reliably and a formal included-versus-excluded missingness model is not possible. We therefore report these statistics as an empirical selection-footprint characterization, not as evidence that missingness is ignorable (Supplementary Tables S3 and S7).

### 3.2. Population-Growth Forecast Performance

Across the feature specifications, momentum plus non-health predictors had the lowest observed RMSE (0.20491), followed by momentum only (0.20503), the full DPC-GB feature set (0.20531), and momentum plus health predictors (0.20680). Momentum only also had the lowest observed MAE (0.13142). Thus, the full model did not achieve the lowest error across all evaluated specifications (Table 3; Supplementary Table S4). The full model’s RMSE was 6.34% lower than persistence (0.21920), but the country-cluster interval for this gain crossed zero, making the comparison inconclusive across countries. Its lower error relative to the original direct level-forecast benchmarks is specific to those fixed configurations. None of the original eight direct learners beat persistence. In the matched-hyperparameter experiment, direct gradient boosting had RMSE 0.22588 and MAE 0.1476, versus RMSE 0.20531 and MAE 0.1336 for the full correction formulation. The direct-minus-correction RMSE difference was 0.02057 (country-cluster 95% interval −0.00044 to 0.04383); the result is consistent with a role for target formulation but is not conclusive.

**Figure 2:**
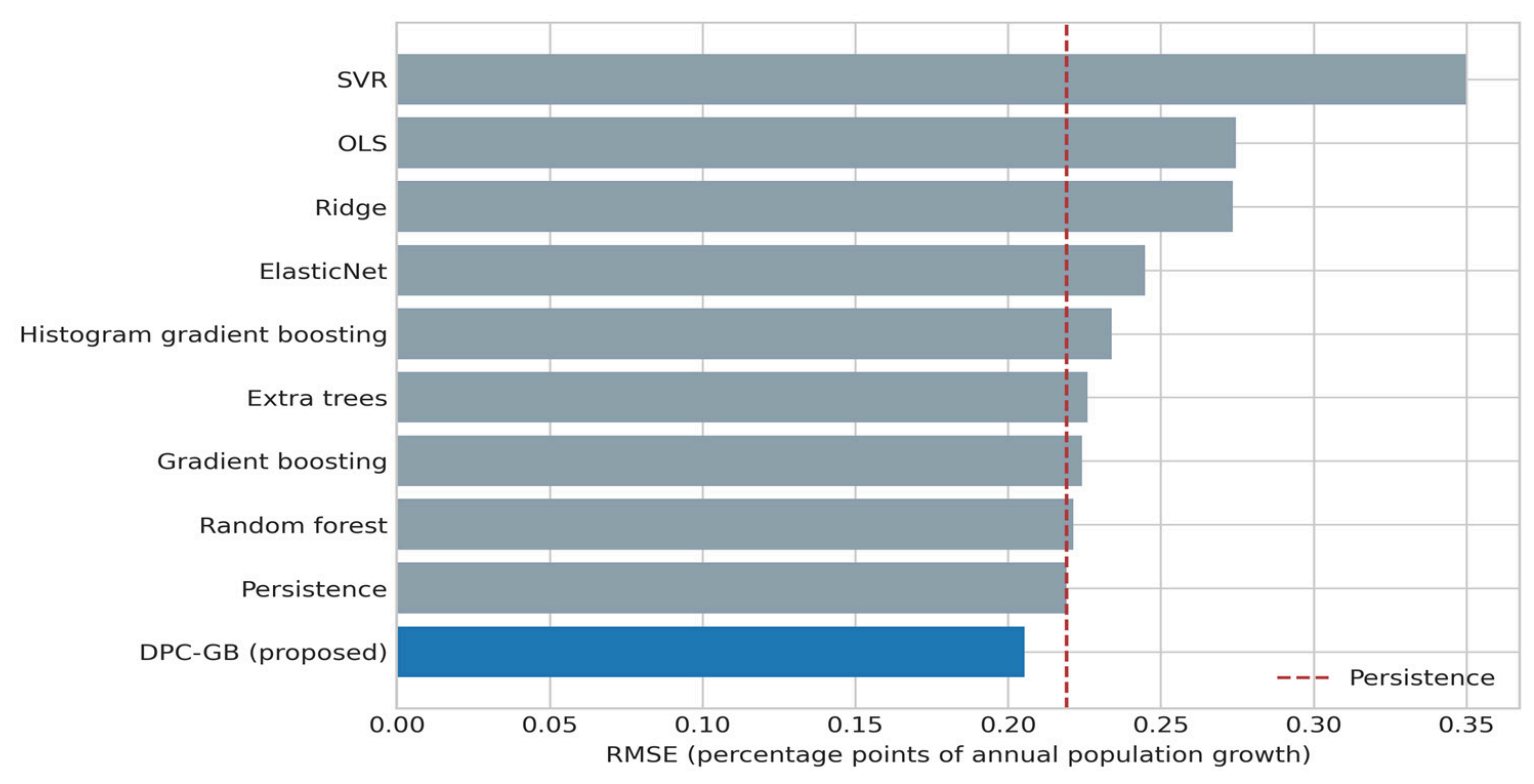
Rolling-origin out-of-sample RMSE for the original ten forecasting approaches.

MAE and RMSE are in percentage points of annual population growth. Rows are ordered by observed RMSE. Positive values in the final column denote lower error than persistence. The original ten-approach benchmark is retained in Supplementary Table S2 and Figure 2; the complete feature-set comparison is in Supplementary Table S4. The very small differences among the leading correction specifications are descriptive and do not establish superiority. R^2^ is dominated by between-country variance (Section 2.6).

The dashed line marks persistence (RMSE = 0.2192). Full DPC-GB is the only correction specification in this original ten-approach plot. The plot does not include momentum-only or momentum-plus-non-health ablations, both of which have lower observed RMSE than the full specification (Table 3; Supplementary Table S4). Errors are percentage points of annual population growth.

The performance advantage was not uniform across countries. The original country-cluster bootstrap estimated a mean absolute RMSE gain of 0.0133 percentage points, with a 95% percentile interval from −0.0038 to 0.0366; the one-sided tail probability for no gain was 0.0968. The primary comparison is therefore inconclusive across countries and is described that way in the Abstract and Conclusions. Excluding the 2020 stress year remained directionally favourable but does not resolve the cross-country uncertainty.

### 3.3. Event-Count Limitation of the Exploratory Onset Analysis

Among 167 outer forecasts, 136 had nonnegative growth at t and were eligible for incident-onset assessment; only one crossed into negative growth at t + 1. This event count cannot validate probability calibration, discrimination or operating thresholds. Probability diagnostics are therefore retained only as exploratory material in the separate Supplementary Materials (Sections S1 and S2, Tables S6 and S8, and Figure S1), without supporting an early-warning performance claim.

### 3.4. What Changes the Persistence Forecast?

**Figure 3:**
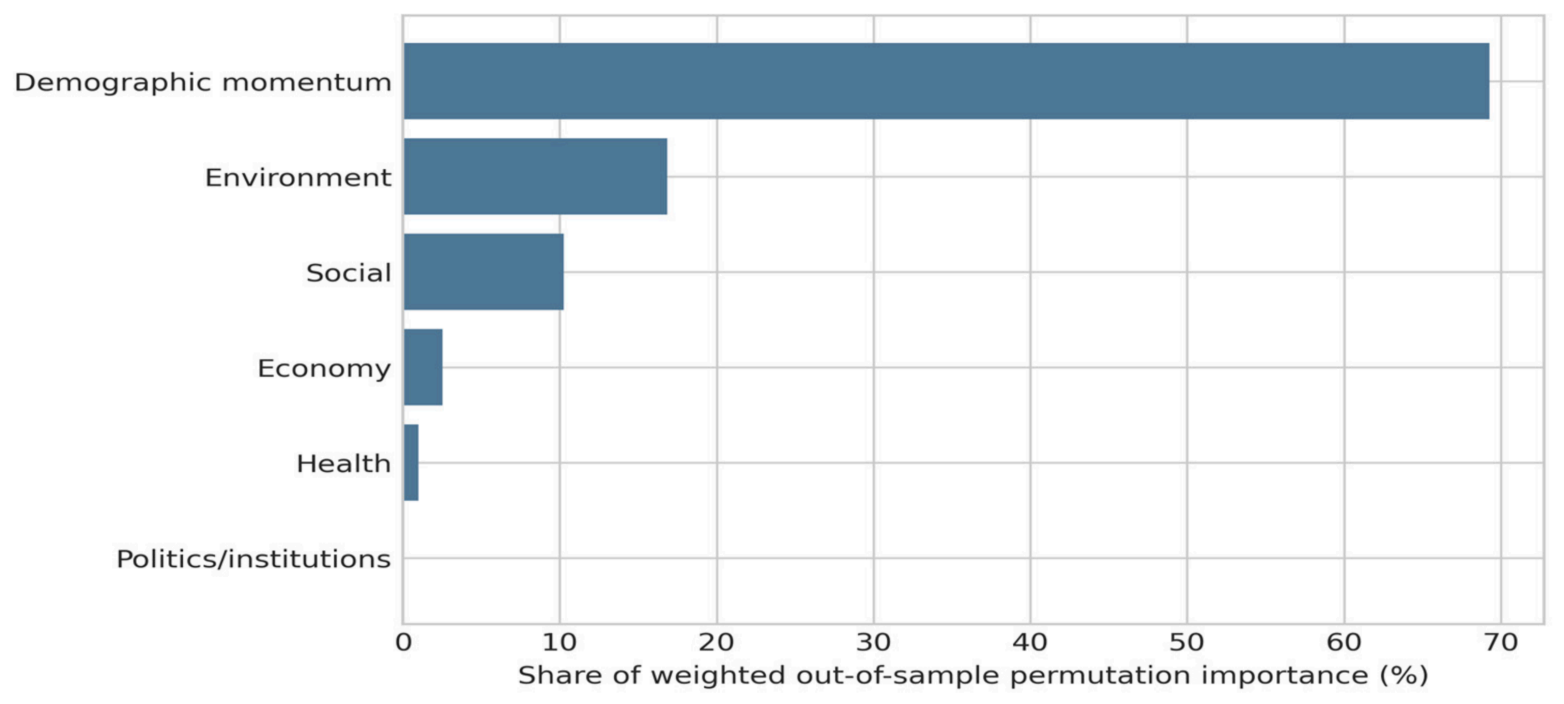
Grouped out-of-sample permutation importance for the learned correction to persistence.

Weighted out-of-sample permutation importance showed that demographic momentum dominated the learned correction, accounting for 69.3% of total importance, as shown in Figure 3. These values describe predictive reliance rather than causal effects and can be redistributed among correlated predictors. Exploratory local SHAP diagnostics showed limited stability: across 99 adjacent country-year pairs, the mean top-three-feature Jaccard overlap was 0.54 (median 0.50). Among 136 onset-eligible forecasts, the most frequent largest local contributor was recent population-growth change (79 forecasts), followed by unemployment change (34) and lagged population growth (15). These diagnostics describe the full correction model and do not validate incident-risk probabilities or establish causal explanations.

Bars show each domain’s share of the weighted deterioration in correction RMSE after feature permutation. Values indicate model reliance, not causal effects.

The structural-only model produced a different ranking because demographic history was removed. Hospital-bed density (beds per 1000 people) ranked highly within that structural-only model, but the model itself forecast poorly (RMSE 0.4582, MAE 0.3597, R^2^ 0.6672) compared with persistence (RMSE 0.2192). The ranking therefore should not be interpreted as evidence that hospital-bed density is a strong population-growth predictor; it only identifies reliance within a weak structural-only specification.

### 3.5. Transfer to Public-Health Planning Endpoints

Transfer analysis produced endpoint-specific results. DPC-GB reduced fertility RMSE from 0.05423 to 0.04277 (21.1%; unadjusted country-cluster interval 0.00552–0.01780) and hospital-bed-density RMSE from 0.11399 to 0.10037 (12.0%; interval 0.00008–0.02842). After Holm adjustment across the four exploratory endpoints, fertility remained supported (adjusted *p* < 0.001), whereas hospital bed density did not meet the conventional 0.05 threshold (adjusted *p* = 0.072). Health expenditure and DPT coverage showed no improvement (adjusted *p* = 1.000 for each). The full transfer results are reported in Table 4.

**Figure 4:**
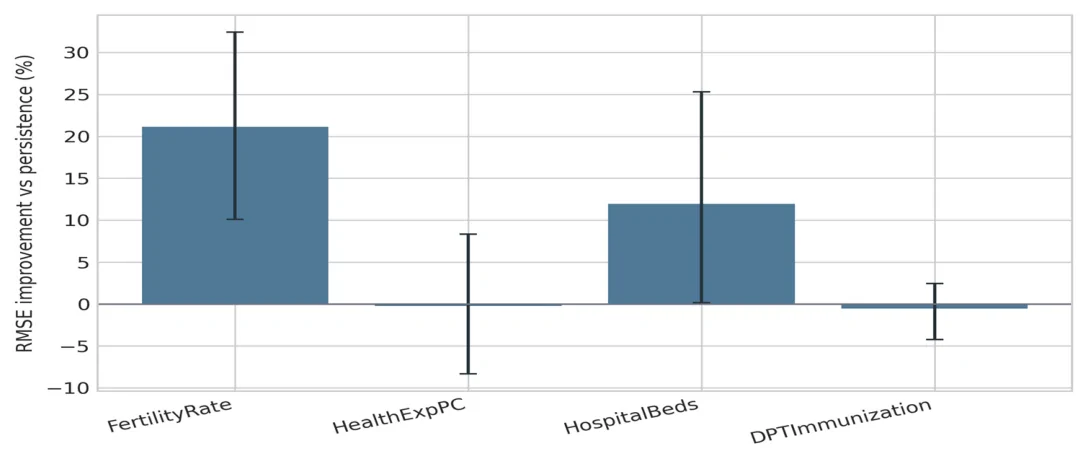
RMSE improvement of DPC-GB relative to persistence for four secondary planning indicators.

Intervals are country-cluster bootstrap percentile intervals for the absolute reduction in RMSE. Holm-adjusted *p* values control multiplicity across the four exploratory endpoints. HospitalBeds denotes hospital-bed density (beds per 1000 people), not absolute bed capacity; the near-zero lower interval bound and adjusted *p* = 0.072 preclude a firm positive conclusion. Figure 4 summarizes the RMSE improvement of DPC-GB relative to persistence across the four secondary planning indicators.

Whiskers show unadjusted country-cluster 95% percentile intervals. Multiplicity-adjusted inference supports fertility only; the hospital-bed-density result is suggestive but not conclusive after Holm correction.

The secondary-endpoint analysis therefore provides evidence for transfer to fertility forecasting but not a firm multiplicity-adjusted conclusion for hospital-bed density, health expenditure or DPT coverage. Absolute bed numbers and population-adjusted service need were unavailable and should be modelled separately before any capacity-planning claim is made.

### 3.6. Health-Information Ablation

The observed RMSE ranking was momentum plus non-health (0.20491), momentum only (0.20503), full DPC-GB (0.20531), momentum plus health (0.20680), and health-only correction (0.24860). Adding health variables did not improve the primary point forecast once momentum was available. The full 42-feature model was 0.00028 RMSE (approximately 0.14%) worse than momentum only. Momentum plus non-health used 34 features and was only 0.00012 RMSE (approximately 0.06%) better than momentum only. These numerical differences do not establish superiority among the leading specifications. For routine one-year forecasting, the three-feature momentum-only model provides a parsimonious option. Cross-domain inputs remain useful for exploring model reliance and separately evaluated endpoints, but the primary accuracy results do not justify their additional data burden (Table 5; Supplementary Table S4).

All five specifications are shown in ascending RMSE order. Negative values in the third column denote lower error than momentum only. Specifications use the identical rolling-origin design and 167 outer forecasts; Supplementary Table S4 additionally reports MAE and R^2^. The ranking is descriptive, and no pairwise superiority claim is made from the small RMSE differences among the leading specifications.

## 4. Discussion

### 4.1. Principal Findings

This study evaluates persistence correction for one-year population-growth forecasting as a potential upstream health-system input. The full DPC-GB model lowered observed aggregate error relative to persistence, but country-cluster uncertainty makes that comparison inconclusive. Momentum plus non-health predictors had the lowest observed RMSE, followed by momentum only and then the full model; the small differences do not establish superiority among these specifications. Health inputs added no primary short-horizon accuracy once momentum was available. With only one incident onset among 136 eligible forecasts, the supplementary probability analysis cannot establish calibration, discrimination or threshold performance.

A central negative finding is that none of the original eight conventional learners applied directly to the growth-rate level outperformed persistence. A matched-hyperparameter experiment further showed lower error for correction targeting than for direct level targeting, but its country-cluster confidence interval still crossed zero. Thus the data are consistent with an advantage of correction formulation, while not establishing a general performance gain across countries.

The secondary analyses define the healthcare relevance more narrowly than in the original manuscript. The primary model predicts demography, not utilization, workforce, expenditure or physical capacity. After multiplicity adjustment, transfer evidence remained for fertility only. Hospital-bed density (beds per 1000 people) cannot substitute for absolute bed stock or need-adjusted capacity. The structural-only model was also weak despite assigning high importance to hospital-bed density, reinforcing the distinction between within-model importance and forecast usefulness. An additional practical finding is parsimony: the three-feature momentum-only specification slightly outperformed the 42-feature full model for the primary one-year point forecast. Thus health-system use, if any, is as an externally validated upstream trigger for further planning analysis, not as a direct resource-allocation output.

### 4.2. Implications for Health-System and Public-Health Planning

Population decline can change health-system needs through several channels. Contraction frequently coexists with ageing, which can increase chronic-disease and long-term-care needs even as the total population falls [1,2,3,4]. At the local level, shrinking populations can also erode the fiscal and workforce base needed to sustain geographically accessible services. Recent evidence from Chinese cities shows that city shrinkage is associated with altered health-service provision, including hospital beds and physicians, and raises equity concerns when supply does not adjust appropriately to population change [5].

From a health-economics perspective, contraction changes both service demand and the financing base. Some costs are fixed or geographically indivisible, while the contribution and tax base may shrink and the per capita burden of maintaining minimum access can rise [7]. Demographic change is therefore relevant to financing and equity decisions [2,6]. The present evidence supports continued evaluation of demographic point forecasts as planning inputs; it does not validate incident-risk probabilities as early-warning triggers.

The present framework is a retrospective proof-of-concept requiring external validation before planning use. A demographic signal could eventually prompt a review of service accessibility or workforce needs, but such review requires age-specific morbidity, utilization, geographic access, workforce, absolute bed counts and budget data. The current study cannot determine resource allocations or support a probability-based alert rule.

### 4.3. Interpreting Structural Health Signals Without Causal Overreach

Hospital-bed density and fertility emerged as leading variables within the structural-only attribution analysis, but the structural-only model had RMSE 0.4582 and was much less accurate than persistence. Accordingly, importance in that model should not be confused with useful forecast performance. In addition, bed density can increase mechanically as population falls. Absolute bed counts, age- and morbidity-adjusted service need, utilization and geographic access are required before drawing capacity-planning conclusions.

Permutation importance should be read as a statement about how fitted predictions depend on available information, not as an intervention effect. Increasing hospital beds would not change population growth by an amount implied by an importance score, and reverse causality is plausible. Causal policy evaluation requires designs with explicit treatments and counterfactual assumptions; the present framework is designed for prediction and early warning [12,23,24].

### 4.4. Event Sparsity and the Scope of Probability Reporting

Restricting assessment to true incident contraction leaves only one event among 136 eligible forecasts. Calibration, discrimination and threshold performance cannot be validated from this event count, regardless of apparently favourable summary metrics. We therefore retain detailed probability results only in the separate Supplementary Materials. Country-cluster refitting does not create new independent events and cannot resolve this fundamental limitation.

### 4.5. Methodological Implications

The persistence-correction design has a general advantage when a naive policy baseline is strong. Learning only the forecast correction makes incremental value explicit and makes feature-importance analysis more policy-relevant: each permutation tests information used to depart from ‘next year equals this year’. The same structure can be applied to other persistent planning indicators, but performance must be validated separately for every endpoint.

Temporal validation is important but does not by itself reproduce the information set available at historical forecast dates. The one-year-lagged structural-covariate sensitivity produced RMSE 0.2043 versus 0.2192 for persistence, with a country-cluster gain interval of −0.0029 to 0.0381 (one-sided *p* = 0.0799), suggesting that the main pattern is not entirely dependent on contemporaneous structural values while remaining inconclusive across countries. Separately, five-fold country-blocked rolling-origin validation held each test-country group out of all training years and produced RMSE 0.2055 versus 0.2192 for persistence (cluster interval for the gain 0.0003–0.0308). This is evidence of within-dataset transfer to held-out jurisdictions, not external validation in a new source system or population.

### 4.6. Limitations

First, complete-case selection reduced the documented source pool from 4616 to 672 observations. The retained-country profile is visibly selective: 74.5% are high-income and 70.2% are in Europe and Central Asia, with no low-income country and only one lower-middle-income country; complete-case coverage is also denser in later years. This pattern is consistent with availability-driven selection linked to statistical/reporting infrastructure. Because the original row-level pre-merge file and upstream merge code were not retained, we cannot directly compare the 3944 excluded rows with the included rows on population size, health expenditure or population-growth values, and we therefore do not claim a formal missingness mechanism. Supplementary Table S7 reports the empirical selection footprint of the retained data. The resulting sample is not globally representative, particularly of low-resource settings where early warning may be most needed. A stronger future design would preserve the full pre-merge panel and perform fold-specific imputation within each training period, with explicit missingness diagnostics and sensitivity analyses [31].

Second, the primary horizon is one year. This horizon is relevant for annual budgeting and near-term service planning, but it is not a substitute for three-, five- or ten-year health-system planning. Persistence is likely to weaken at longer horizons, and age–sex structure, births, deaths and migration components become correspondingly more important. External multi-horizon validation is therefore a priority.

Third, the dataset does not include age distribution, cause-specific morbidity, health-care utilization, clinician workforce, waiting times, geographic accessibility or health outcomes such as mortality and disability. It also lacks absolute hospital-bed counts and age- or morbidity-adjusted service need. Hospital-bed density (beds per 1000 people) is a ratio whose population denominator can fall; the measure therefore cannot be equated with physical capacity. Country-level aggregates further cannot address the sub-national scale at which many capacity decisions are made.

Fourth, only one incident contraction occurred among 136 eligible outer forecasts. Probability calibration, discrimination and operating thresholds consequently remain unvalidated. The exploratory residual-based procedure also does not explicitly model country-specific heteroskedasticity, tails or regime shifts. Cluster refitting propagates fitting and residual-construction uncertainty but cannot increase the number of independently observed events. Larger panels with more incident transitions and prospective vintage-aware evaluation are required before probability-based planning use.

Fifth, the full model’s observed RMSE was 6.34% lower than persistence, but its country-cluster difference interval included zero. Momentum plus non-health and momentum only both had lower observed RMSE than the full model, although their small numerical differences do not establish superiority. Hyperparameters were fixed rather than selected by nested optimization, so algorithm rankings are configuration-specific. The matched direct-versus-correction interval also crossed zero. Country-blocked validation does not substitute for external validation. After Holm correction, transfer evidence remained for fertility only; hospital-bed density was suggestive (adjusted *p* = 0.072), and health expenditure and DPT coverage showed no improvement.

Finally, data provenance is incomplete upstream of the merged analytical file. Supplementary Table S1 now reports the retained merged-file identifiers, analytical units/scales and source organizations, and Section 2.1 documents the integrity checks applied to the retained merged file and dynamic panel. However, variable-specific upstream agency series identifiers, original download dates/source vintages and the original cross-agency merge code are not available in the retained materials. The provenance audit therefore begins at the retained merged-file boundary and does not reconstruct unavailable upstream history.

## 5. Conclusions

Persistence correction warrants further evaluation for short-horizon demographic forecasting as a possible upstream input to health-system planning. The full DPC-GB model had 6.34% lower observed RMSE than persistence, but the country-cluster interval included zero, so improved forecasting across countries was not established. Momentum plus non-health predictors had the lowest observed RMSE (0.20491), followed by momentum only (0.20503) and the full model (0.20531); the small differences do not establish superiority, and momentum only offers a parsimonious three-feature option. Complete-case selection limits generalizability. Only one incident contraction occurred among 136 eligible forecasts, preventing validation of calibration, discrimination or operating thresholds; probability diagnostics are supplementary and exploratory. After multiplicity correction, transfer evidence remained for fertility only. Potential health-system use depends on external validation and linkage to actual utilization, workforce, morbidity, access and physical-capacity data.

## Figures and Tables

**Table 1:** Structured comparison with closely related demographic-forecasting and health-planning approaches.

Approach	Representative Studies	Target/Evaluation Emphasis	Difference from the Present Study
Stochastic/Bayesian population projection	Lee & Tuljapurkar [8]; Raftery et al. [9,10,11]	Long-horizon population distributions, often by age/sex and demographic components; explicit probabilistic uncertainty.	Provides the core probabilistic-demography benchmark; the present study focuses on one-year persistence correction, with incident-onset diagnostics confined to exploratory Supplementary Materials.
Machine-learning population forecasting	Sahinarslan et al. [13]	Direct population-level forecasting using multiple ML and time-series algorithms across many countries and demographic indicators.	Focuses on algorithmic level prediction rather than incremental correction to a strong persistence baseline.
Multi-source subnational ML forecasting	Wang et al. [14]	One-year demographic prediction in Hebei using census/mobile data and tuned SSA-XGBoost; algorithm and optimization comparisons.	Uses optimized high-dimensional local data; does not evaluate cross-country incident contraction or health-information ablation.
Health-system resource projection	Rachet-Jacquet et al. [2]	Long-term healthcare demand, funding, beds and workforce based on projected population structure and utilization.	Directly models health-system resources; the present primary model instead forecasts demography as a possible upstream planning signal.
Present study	DPC-GB framework	One-year population-growth correction; rolling-origin validation, matched formulation test, cluster uncertainty, ablation and transfer analyses. Incident-onset diagnostics are supplementary and exploratory.	Evaluates incremental accuracy beyond persistence and limits of demographic forecasts as upstream health-planning inputs; probability performance is not validated.

**Table 2:** Study sample and validation design.

Item	Value
Documented source pool	4616 country-year observations (2000–2020)
Complete-case analytical file	672 observations; 47 countries
Consecutive dynamic panel	522 observations; 45 countries
Outer rolling-origin target years	2016–2020
Outer out-of-sample forecasts	167
At-risk onset forecasts: PopulationGrowth(t) ≥ 0	136
Observed incident contractions: PopulationGrowth(t + 1) < 0	1 of 136 (0.7%)
Monte Carlo draws per forecast (supplementary exploratory analysis)	10,000
Country-cluster point-forecast bootstrap replicates	10,000
Full-pipeline refits (supplementary exploratory analysis)	500

**Table 3:** Selected benchmark models and leading feature specifications for one-year-ahead population-growth forecasts (*n* = 167).

Model	MAE	RMSE	R^2^	RMSE Improvement vs. Persistence
DPC-GB: momentum + non-health	0.13385	0.20491	0.9334	+6.52%
DPC-GB: momentum only	0.13142	0.20503	0.9334	+6.46%
DPC-GB: full feature set	0.13362	0.20531	0.9332	+6.34%
Persistence (benchmark)	0.1420	0.2192	0.9238	—
Random forest	0.1478	0.2214	0.9223	−1.00%
Gradient boosting	0.1469	0.2242	0.9203	−2.28%
Extra trees	0.1481	0.2260	0.9190	−3.12%
Histogram gradient boosting	0.1578	0.2340	0.9132	−6.74%
OLS	0.1897	0.2746	0.8805	−25.26%

**Table 4:** Transfer of the DPC-GB architecture to four public-health planning indicators.

Endpoint	Persistence RMSE	DPC-GB RMSE	RMSE Gain	Bootstrap 95% CI (RMSE Units)	Holm-Adjusted *p*
Total fertility rate	0.05423	0.04277	+21.1%	[0.00552, 0.01780]	<0.001
Hospital-bed density (beds per 1000 people)	0.11399	0.10037	+12.0%	[0.00008, 0.02842]	0.072
Log health expenditure per capita	0.07359	0.07376	−0.2%	[−0.00612, 0.00614]	1.000
DPT immunization coverage	0.02600	0.02614	−0.5%	[−0.00112, 0.00064]	1.000

**Table 5:** Complete health-information ablation and feature burden for the primary population-growth outcome.

Feature Set	RMSE	Δ vs. Momentum-Only	RMSE Improvement vs. Persistence	No. Features
Momentum + non-health	0.20491	−0.00012	+6.52%	34
Momentum only	0.20503	—	+6.46%	3
Full DPC-GB feature set	0.20531	+0.00028	+6.34%	42
Momentum + health	0.20680	+0.00176	+5.66%	11
Health-only correction	0.24860	+0.04356	−13.41%	8

## Data Availability

The retained analysis-ready merged data, data dictionary, processed panel, executable analysis code, outer predictions, derived tables, figures, environment specifications and checksums are publicly available at https://github.com/Nahj5525/machine-learning-for-early-warning-of-demographic-contraction (accessed on 14 October 2026). The version cited for this manuscript is main-branch commit 3442a32c485209b1f63c1654df59ac33f20598ad (14 October 2026), containing Reproducibility_Package(Demographic Contraction for Health-System Planning).zip. These materials reproduce the analysis from the retained merged dataset forward. The merged-file variable identifiers, analytical units/scales and source organizations are documented, but variable-specific upstream source-series identifiers, original download dates/source vintages and the original cross-agency extraction/merge code were not retained and cannot be reconstructed reliably; the repository should therefore not be interpreted as a complete reconstruction of upstream data acquisition.

## Supplementary Materials

The following supporting information can be downloaded at: https://www.mdpi.com/article/10.3390/healthcare14203494/s1, Table S1: Retained merged-file identifiers, variable definitions, analytical units/scales and provenance status; Table S2: Complete ten-model benchmark, full hyperparameter configurations and prespecification rationale; Table S3: Countries in the complete-case file and dynamic panel, with region and income group; Table S4: Complete health-information ablation for the primary population-growth outcome; Table S5: Reviewer-requested robustness analyses isolating formulation, timing and jurisdiction transfer; Table S6: Exploratory incident-onset probability diagnostics from 500 full-pipeline country-cluster refits; Table S7: Empirical characterization of the complete-case selection footprint; Table S8: Exploratory incident-contraction threshold metrics (136 eligible forecasts; one incident event); Sections S1 and S2: Exploratory methods for incident-onset probability diagnostics and exploratory results and event-count diagnostics; Figure S1: Exploratory incident-contraction probability and event-count diagnostics. Retained reproducibility materials are available at https://github.com/Nahj5525/machine-learning-for-early-warning-of-demographic-contraction (commit 3442a32c485209b1f63c1654df59ac33f20598ad, 14 October 2026); upstream provenance limitations are stated in the Data Availability Statement and Supplementary Table S1.
