# Supplementary material for: Predicting Demographic Contraction as an Upstream Signal for Health-System Planning: A Persistence-Corrected Machine-Learning Framework
Source: Healthcare (Basel). 2026 Oct 17;14(20):3494. doi: 10.3390/healthcare14203494 (PMC13704099; doi:10.3390/healthcare14203494)
Supplement: Supplementary file 1 [file healthcare-14-03494-s001.zip › healthcare-4579083-supplementary.pdf]

Supplementary Materials

Predicting Demographic Contraction as an Upstream Signal for Health-System Planning: A Persistence-Corrected Machine-Learning Framework

This separate supplement accompanies the second-round revised manuscript. Tables S1–S7 retain the preceding revision's numerical results. Sections S1 and S2, Table S8 and Figure S1 preserve incident-onset analyses solely for exploratory transparency. Only one incident event occurred among 136 eligible forecasts; probability calibration, discrimination and operating thresholds cannot be validated. Yellow highlighting identifies revised labels and added or revised explanatory text.

Supplementary Table S1. Retained merged-file identifiers, variable definitions, analytical units/scales and provenance status.

Domain	Retained identifier	Definition	Analytical unit / stored scale	Source
Outcome	PopulationGrowth	Annual population growth rate	percentage points per year	World Bank
Health	FertilityRate	Total fertility rate	births per woman	World Bank
Health	HealthExpPC	Per-capita health expenditure, natural-log transformed	ln(US$ per capita)	World Bank
Health	HospitalBeds	Hospital-bed density	beds per 1,000 people	WHO
Health	DPTImmunization	Infant DPT immunization coverage	proportion (0–1; source percentage rescaled)	WHO
Economy	GDPpc	GDP per capita at current prices, natural-log transformed	ln(current US$ per capita)	IMF
Economy	GDPWorldShare	Domestic GDP as a share of world output	percentage points of world GDP	IMF
Economy	CPIGrowth	Consumer price index growth rate	proportion per year (source percentage rescaled)	IMF
Economy	GDPDeflator	GDP deflator, natural-log transformed	ln(source index value)	IMF
Social	PrimaryExpGDP	Primary-education expenditure relative to GDP	percentage points of GDP	OECD
Social	TertiaryExpGDP	Tertiary-education expenditure relative to GDP	percentage points of GDP	OECD
Social	YouthUnempRate	Youth unemployment rate	proportion (0–1; source percentage rescaled)	World Bank
Social	UnempRate	Total unemployment rate	proportion (0–1; source percentage rescaled)	World Bank
Politics / institutions	CivilSocietyIndex	Core Civil Society Index	index, 0–1 (higher = stronger/more independent civil society)	V-Dem
Politics / institutions	EqualProtection	Equal Protection Index	index, 0–1 (higher = greater equal protection)	V-Dem
Politics / institutions	FreedomExpression	Freedom of Expression and Alternative Sources of Information	index, 0–1 (higher = greater freedom)	V-Dem
Politics / institutions	AccessJustice	Access to Justice for All Social Groups	index, 0–1 (higher = greater access)	V-Dem
Environment	RenewEnergyShare	Renewable-energy consumption share	proportion (0–1; source percentage rescaled)	World Bank
Environment	FreshwaterPC	Per-capita renewable internal freshwater resources, natural-log transformed	ln(source per-capita freshwater measure)	World Bank
Environment	GHGEmissions	Greenhouse-gas emissions, natural-log transformed	ln(source-reported emissions measure)	OECD
Environment	AirPollutionExposure	Annual air-pollution exposure, natural-log transformed	ln(source-reported exposure measure)	OECD

Note. The identifier column reports the verbatim variable names retained in the merged analytical file, and the unit/scale column reports the stored analytical scale. The merged file is the authoritative basis for these scales. DPT coverage, unemployment rates and renewable-energy share are stored as 0–1 proportions. Source organizations were retained in the supplied dictionary. No variable-specific upstream agency series identifier, original download date, source vintage, or original cross-agency merge code could be recovered from the retained materials; none is reconstructed or inferred. Thus, the table provides a retained-file provenance audit rather than a complete upstream source-series audit trail.

Supplementary Table S2. Complete ten-model benchmark, full hyperparameter configurations and prespecification rationale.

Panel A. Rolling-origin out-of-sample performance (n = 167)

Model	N	MAE	RMSE	R^2	RMSE improvement vs persistence
DPC-GB (proposed)	167	0.1336	0.2053	0.9332	+6.34%
Persistence	167	0.1420	0.2192	0.9238	—
RandomForest	167	0.1478	0.2214	0.9223	−1.00%
GradientBoosting	167	0.1469	0.2242	0.9203	−2.28%
ExtraTrees	167	0.1481	0.2260	0.9190	−3.12%
HistGradientBoosting	167	0.1578	0.2340	0.9132	−6.74%
ElasticNet	167	0.1798	0.2448	0.9050	−11.69%
Ridge	167	0.1899	0.2736	0.8813	−24.81%
OLS	167	0.1897	0.2746	0.8805	−25.26%
SVR	167	0.2368	0.3499	0.8059	−59.64%

Note. MAE and RMSE are in percentage points of annual population growth. Positive improvement denotes lower RMSE than persistence. The ten evaluated approaches comprise persistence, DPC-GB, and eight direct level-forecast learners.

Panel B. Full model configurations

Model	Configuration
Persistence	No fitted parameters; forecast = current population growth.
OLS	StandardScaler + ordinary least squares (LinearRegression; default fit intercept).
Ridge	StandardScaler + Ridge(alpha = 1.0).
ElasticNet	StandardScaler + ElasticNet(alpha = 0.01, l1_ratio = 0.5, max_iter = 10,000, random_state = 20260817).
SVR-RBF	StandardScaler + SVR(kernel = RBF, C = 10, epsilon = 0.05, gamma = scale).
RandomForest	RandomForestRegressor(n_estimators = 300, min_samples_leaf = 2, max_features = 0.8, random_state = 20260817, n_jobs = -1).
ExtraTrees	ExtraTreesRegressor(n_estimators = 300, min_samples_leaf = 2, max_features = 0.8, random_state = 20260817, n_jobs = -1).
GradientBoosting	GradientBoostingRegressor(n_estimators = 150, max_depth = 2, learning_rate = 0.03, loss = Huber, random_state = 20260817).
HistGradientBoosting	HistGradientBoostingRegressor(max_iter = 200, max_leaf_nodes = 15, l2_regularization = 1.0, learning_rate = 0.05, random_state = 20260817).
DPC-GB (proposed)	Persistence anchor + GradientBoostingRegressor on next-year correction (n_estimators = 80, max_depth = 2, learning_rate = 0.05, loss = Huber, random_state = 20260817).
Matched direct GradientBoosting (Reviewer 1 sensitivity)	GradientBoostingRegressor(n_estimators = 80, max_depth = 2, learning_rate = 0.05, loss = Huber, random_state = 20260817), fitted directly to the t+1 level.

Note. All outer test years were 2016–2020 using expanding-window rolling-origin validation. Hyperparameters were fixed before evaluation and were not selected by optimizing performance on the outer test years. DPC-GB used a deliberately low-capacity depth-2 gradient-boosting configuration with shrinkage and Huber loss, selected by expert judgment for a modest panel; competing models used conventional fixed benchmark configurations with standard regularization or ensemble sizes. No nested hyperparameter search was performed, so cross-algorithm rankings are

descriptive for these prespecified configurations. Linear, ridge, elastic-net and SVR inputs were standardized. DPC-GB was fitted to Target(t+1) − PopulationGrowth(t), while the matched direct GradientBoosting sensitivity used the identical DPC-GB estimator settings but predicted Target(t+1) directly.

Supplementary Table S3. Countries in the complete-case file and dynamic panel, with region and income group.

Country	ISO3	Dynamic panel	World Bank region	Income group (FY2021)
Greece	GRC	Yes	Europe & Central Asia	High income
South Africa	ZAF	No	Sub-Saharan Africa	Upper middle income
Netherlands	NLD	Yes	Europe & Central Asia	High income
Norway	NOR	Yes	Europe & Central Asia	High income
New Zealand	NZL	Yes	East Asia & Pacific	High income
Korea, Rep.	KOR	Yes	East Asia & Pacific	High income
Denmark	DNK	Yes	Europe & Central Asia	High income
Germany	DEU	Yes	Europe & Central Asia	High income
Latvia	LVA	Yes	Europe & Central Asia	High income
Romania	ROU	Yes	Europe & Central Asia	High income
Luxembourg	LUX	Yes	Europe & Central Asia	High income
Mexico	MEX	Yes	Latin America & Caribbean	Upper middle income
Malta	MLT	Yes	Europe & Central Asia	High income
United States	USA	Yes	North America	High income
Belgium	BEL	Yes	Europe & Central Asia	High income
Bulgaria	BGR	Yes	Europe & Central Asia	Upper middle income
Brazil	BRA	Yes	Latin America & Caribbean	Upper middle income
Sweden	SWE	Yes	Europe & Central Asia	High income
Switzerland	CHE	Yes	Europe & Central Asia	High income
Spain	ESP	Yes	Europe & Central Asia	High income
Slovak Republic	SVK	Yes	Europe & Central Asia	High income
Slovenia	SVN	Yes	Europe & Central Asia	High income
Argentina	ARG	Yes	Latin America & Caribbean	Upper middle income
Iceland	ISL	Yes	Europe & Central Asia	High income
Ireland	IRL	Yes	Europe & Central Asia	High income
Estonia	EST	Yes	Europe & Central Asia	High income
United Kingdom	GBR	Yes	Europe & Central Asia	High income
Australia	AUS	Yes	East Asia & Pacific	High income
Austria	AUT	Yes	Europe & Central Asia	High income
Ukraine	UKR	Yes	Europe & Central Asia	Lower middle income
Israel	ISR	Yes	Middle East & North Africa	High income
Italy	ITA	Yes	Europe & Central Asia	High income
Indonesia	IDN	Yes	East Asia & Pacific	Upper middle income
Czechia	CZE	Yes	Europe & Central Asia	High income
Chile	CHL	Yes	Latin America & Caribbean	High income
Kazakhstan	KAZ	Yes	Europe & Central Asia	Upper middle income
Costa Rica	CRI	Yes	Latin America & Caribbean	Upper middle income
Colombia	COL	Yes	Latin America & Caribbean	Upper middle income
Croatia	HRV	No	Europe & Central Asia	High income
Cyprus	CYP	Yes	Europe & Central Asia	High income
Türkiye	TUR	Yes	Europe & Central Asia	Upper middle income
Peru	PER	Yes	Latin America & Caribbean	Upper middle income
Portugal	PRT	Yes	Europe & Central Asia	High income
Poland	POL	Yes	Europe & Central Asia	High income
France	FRA	Yes	Europe & Central Asia	High income
Finland	FIN	Yes	Europe & Central Asia	High income
Hungary	HUN	Yes	Europe & Central Asia	High income

Note. All 47 countries listed are present in the complete-case analytical file. "Dynamic panel = Yes" denotes inclusion among the 45 countries with at least one consecutive t−1,t,t+1 observation. South Africa and Croatia are the two complete-case countries absent from the dynamic panel. Regions follow the World Bank regional taxonomy. Income group is the World Bank FY2021 analytical classification (published for the study end-period; based on 2019 GNI per capita). Region and income group are descriptive only and were not model predictors.

Supplementary Table S4. Complete health-information ablation for the primary population-growth outcome.

Feature set	N	No. features	MAE	RMSE	R^2	Δ RMSE vs momentum only	RMSE improvement vs persistence
Momentum only	167	3	0.13142	0.20503	0.9334	—	+6.46%
Momentum + health	167	11	0.13557	0.20680	0.9322	+0.00176	+5.66%
Momentum + non-health	167	34	0.13385	0.20491	0.9334	−0.00012	+6.52%
Full DPC-GB feature set	167	42	0.13362	0.20531	0.9332	+0.00028	+6.34%
Health-only correction	167	8	0.16688	0.24860	0.9020	+0.04356	−13.41%

Note. All specifications use the identical 2016–2020 rolling-origin design and 167 outer forecasts. DPC-GB uses 80 estimators, maximum depth 2, learning rate 0.05, Huber loss, and seed 20260817. Momentum comprises current growth, its lag, and recent change. The leakage-prone d_FreshwaterPC feature is excluded. Observed RMSE is lowest for momentum plus non-health (0.20491), followed by momentum only (0.20503), full DPC-GB (0.20531), momentum plus health (0.20680), and health-only correction (0.24860). These small leading differences do not establish superiority.

Supplementary Table S5. Reviewer-requested robustness analyses isolating formulation, timing and jurisdiction transfer.

Analysis	RMSE: comparator / DPC	Gain [95% cluster interval]	One-sided p	Interpretation
Matched direct vs correction GB	0.22588 / 0.20531	0.02057 [−0.00044, 0.04383]	0.0277	Direction favors correction; two-sided interval crosses zero.
Lagged structural covariates vs persistence	0.21920 / 0.20435	0.01485 [−0.00291, 0.03806]	0.0799	Not a true vintage-data backtest.
Country-blocked rolling-origin vs persistence	0.21920 / 0.20550	0.01370 [0.00032, 0.03075]	0.0200	Within-dataset transfer to held-out countries.
Structural-only GB vs persistence	0.21920 / 0.45819	−0.23899	—	Weak model (MAE 0.35965; R^2 0.66715).

Note. Both matched gradient-boosting models use 80 estimators, depth 2, learning rate 0.05, Huber loss, and seed 20260817. Cluster intervals resample countries. These sensitivity analyses do not replace independent external validation.

Supplementary Table S6. Exploratory incident-onset probability diagnostics from 500 full-pipeline country-cluster refits.

Quantity	Point / median	95% cluster-refit interval	Estimability / note
Incident-onset Brier score	0.0074 / 0.0070	[0.0017, 0.0188]	All 500 refits
Calibration intercept	3.10 / 5.07	[−1.80, 410.60]	Estimable in 319/500 refits; severe separation/event sparsity
Calibration slope	6.18 / 15.29	[1.16, 692.87]	Estimable in 319/500 refits; severe separation/event sparsity

Quantity	Point / median	95% cluster-refit interval	Estimability / note
Sensitivity at threshold 0.20	1.000 / 1.000	[0.000, 1.000]	Event-containing refits only for sensitivity
Specificity at threshold 0.20	0.985 / 0.979	[0.938, 1.000]	All 500 refits
PPV at threshold 0.20	0.333 / 0.200	[0.000, 0.500]	466/500 refits had at least one alert
Sensitivity at threshold 0.50	0.000 / 0.000	[0.000, 1.000]	Highly unstable
PPV at threshold 0.50	NE / 0.000	[0.000, 1.000]	Only 43/500 refits had an estimable PPV
Sensitivity at threshold 0.80	0.000 / 0.000	[0.000, 0.000]	Event-containing refits only
PPV at threshold 0.80	NE / 0.000	—	Only 3/500 refits had an estimable PPV
Brier skill vs persistence-residual	0.081 / 0.093	[−0.909, 0.553]	Fixed-sample / median across 500 full-pipeline refits; interval crosses zero
Brier skill vs climatology	0.007 / −0.123	[−104.413, 0.459]	Extremely unstable because many cluster resamples contain no incident event

Note. Only one incident event occurred among 136 eligible forecasts. Cluster refits do not create independent new events; percentile ranges, calibration estimates and threshold quantities are exploratory diagnostics, not validation evidence. Calibration parameters were estimable in only 319 of 500 refits and are unstable under separation. NE denotes not estimable; a degenerate bootstrap range does not imply certainty. Methods and results are described in Sections S1 and S2.

Supplementary Table S7. Empirical characterization of the complete-case selection footprint.

Dimension	Retained complete-case profile	Interpretation
Source-pool retention	672 of 4,616 documented country-year rows retained (14.6%)	Approximately 85% attrition before complete-case analysis.
Region of retained countries	Europe & Central Asia 33/47 (70.2%); Latin America & Caribbean 7/47 (14.9%); East Asia & Pacific 4/47 (8.5%); Sub-Saharan Africa, North America and Middle East & North Africa 1 each	Strong geographic concentration; South Asia is absent.
Income group of retained countries	High income 35/47 (74.5%); upper middle income 11/47 (23.4%); lower middle income 1/47 (2.1%); low income 0/47	Retained countries are strongly concentrated in higher-income settings.
Temporal coverage	19 observations in 2000; maximum 42 in 2017; 231/672 (34.4%) occur in 2015–2020	Complete-case availability is denser in later years.
Population-growth pattern	Median 0.575 percentage points/year; IQR 0.081–1.140; 144/672 (21.4%) negative-growth observations	The retained file contains both growth and contraction but is not a random global sample.
Per-capita health expenditure	Median ln(US$) 7.477; IQR 6.578–8.352; approximately US$1,767 (US$719–4,241) on the pre-log scale	Describes retained values only; excluded-row values are unavailable.
Hospital-bed density	Median 4.1 beds/1,000; IQR 2.8–6.4	Describes retained values only; it is not absolute bed capacity.
Excluded-row comparison	Not directly estimable from retained artifacts	The pre-merge row-level file, upstream source vintages and original merge code were not retained; excluded values are therefore not reconstructed or imputed post hoc.

Note. Region and FY2021 income-group labels follow the classifications already reported in Supplementary Table S3. Population-growth, health-expenditure and bed-density summaries are calculated from the retained 672-row complete-case file. Because the original excluded rows are not preserved as an analyzable pre-merge file, this table

characterizes the observed selection footprint but cannot estimate a formal missingness model or an included-versus-excluded effect.

S1 Exploratory methods for incident-onset probability diagnostics

For each outer test observation, DPC-GB produced a point forecast. Before every outer target year, an inner rolling-origin residual pool was generated using only earlier target years, and 10,000 empirical residual draws were added to each point forecast. For the primary onset analysis, probabilities were evaluated only for observations with PopulationGrowth(t) ≥ 0; the event was PopulationGrowth(t+1) < 0. This distinction separates incident contraction from persistence of an already negative growth rate. Two probability baselines were retained: a persistence-residual forecast based on historical next-year changes and expanding-window onset climatology.

Discrimination and calibration were treated as distinct properties. ROC-AUC and average precision assess ranking/discrimination, whereas calibration asks whether predicted probabilities agree with observed event frequencies; high discrimination therefore does not imply adequate calibration [27]. Probability performance was summarized primarily with the Brier score and Brier skill relative to the persistence-residual and expanding-window climatology references. Calibration intercept and slope and year-specific Brier scores were examined where estimable; ROC-AUC and average precision were reported only descriptively because the incident-onset sample contains only one event. To propagate country dependence through model fitting and probability construction, we performed 500 country-cluster bootstrap resamples in which countries were resampled, the rolling DPC-GB models were refitted, historical residual pools were rebuilt with the sampled clusters, and onset probabilities were recalculated. With only one observed incident event in the at-risk outer sample, calibration parameters remain weakly identified even under this resampling scheme.

Illustrative probability thresholds of 0.20, 0.50 and 0.80 were chosen to describe the retained outputs, without optimization or operational endorsement. Exact two-sided Clopper–Pearson intervals describe fixed-sample binomial uncertainty and do not account for country dependence. Supplementary Table S6 separately reports 500 full-pipeline country-cluster refits. Neither interval approach can validate threshold performance from one independent incident event.

S2 Exploratory results and event-count diagnostics

Of 167 outer forecasts, 136 had nonnegative population growth at t and therefore entered the incident-onset analysis. Only one of these 136 forecasts crossed into negative growth at t+1 (0 events in 2016–2018, 1 in 2019, and 0 in 2020). The DPC-GB onset Brier score was 0.0074, compared with 0.0081 for the persistence-residual baseline and 0.0075 for expanding-window onset climatology, corresponding to fixed-sample Brier skill of 8.1% and 0.7%, respectively. Year-specific Brier scores were 0.0033 (2016), 0.0072 (2017), 0.0066 (2018), 0.0153 (2019), and 0.0035 (2020). Because 2019 was the only year containing an incident event, year-specific ROC-AUC and conventional calibration parameters were not estimable in four of five target years. With a single positive event overall, aggregate ROC-AUC (0.993) and average precision (0.500) are also not stable measures of general onset discrimination and are not used to support a performance claim.

Supplementary Table S8. Exploratory incident-contraction threshold metrics (136 eligible forecasts; one incident event).

Risk threshold	Sensitivity [95% CI]	Specificity [95% CI]	PPV [95% CI]	NPV [95% CI]	TP / FP / FN
0.20	1.000 [0.025, 1.000]	0.985 [0.948, 0.998]	0.333 [0.008, 0.906]	1.000 [0.973, 1.000]	1 / 2 / 0
0.50	0.000 [0.000, 0.975]	1.000 [0.973, 1.000]	NE	0.993 [0.960, 1.000]	0 / 0 / 1
0.80	0.000 [0.000, 0.975]	1.000 [0.973, 1.000]	NE	0.993 [0.960, 1.000]	0 / 0 / 1

Note. PPV, positive predictive value; NPV, negative predictive value; NE, not estimable because no positive alerts were generated. Brackets show exact two-sided 95% Clopper–Pearson intervals. These descriptive values do not establish validated sensitivity, specificity, precision or an operating cutoff.

At a 0.20 threshold, the single incident event was detected with two false positives. At 0.50 and 0.80, no alert was generated and the incident event was missed. The exact binomial intervals and cluster-refit intervals are both wide, confirming that the three thresholds are descriptive examples from this dataset only. They are neither optimized cutoffs nor candidate operational thresholds, and the available data do not support threshold selection for deployment.

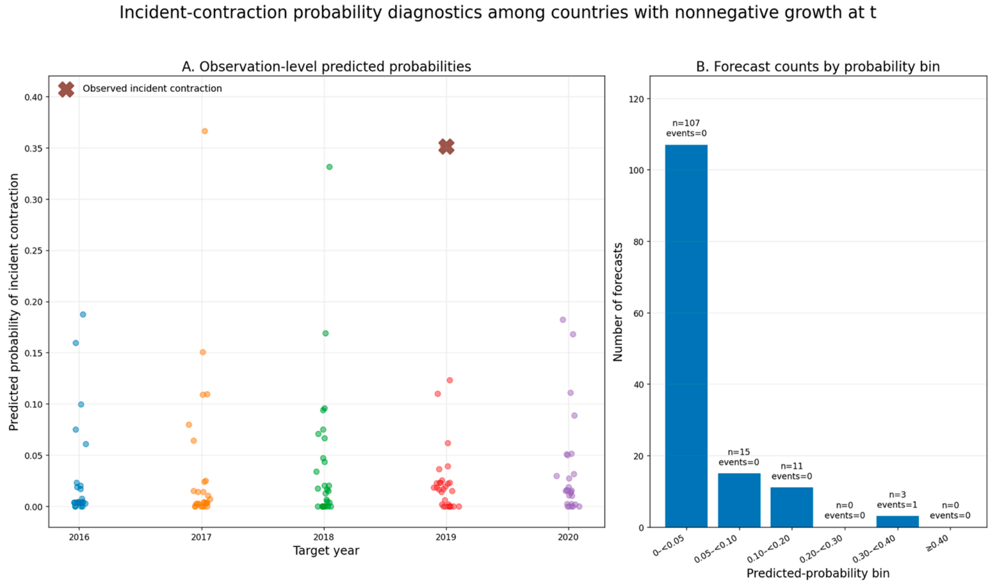

Figure S1. Exploratory incident-contraction probability and event-count diagnostics. Panel A displays observation-level probabilities by target year; Panel B displays forecast counts in probability bins and observed event counts. This is not a reliability curve or evidence of validated calibration.

Panel B makes the extreme sparsity explicit: 107 forecasts fell in [0, 0.05), 15 in [0.05, 0.10), 11 in [0.10, 0.20), none in [0.20, 0.30), 3 in [0.30, 0.40), and none at ≥0.40. The single observed incident fell in the [0.30, 0.40) bin. The figure is a distribution and event-count diagnostic, not a reliability curve or evidence of calibration, because four of five target years contain no incident event.

The reviewer-requested onset restriction changes the interpretation of the probabilistic analysis. The apparent near-perfect discrimination in the original all-state analysis was driven primarily by persistence of already negative growth. Once the task is restricted to true onset among countries with nonnegative current growth, only one event remains. The resulting Brier score is low largely because the base rate is extremely low, while calibration slope/intercept, precision and threshold sensitivity are too weakly identified for firm claims. The probabilistic component is therefore retained as a methodological demonstration, not as validated onset warning.

Full-pipeline country-cluster refitting reinforced this limitation rather than resolving it. Across 500 resamples the Brier-score median was 0.0070 (95% percentile interval 0.0017–0.0188). Brier skill relative to the persistence-residual reference had a median of 0.093 but a 95% interval of −0.909 to 0.553, while the climatology-referenced skill distribution was extremely unstable because many resamples contained no incident event. Calibration intercept/slope were estimable in only 319 resamples and had extremely wide intervals because many resamples contained no event or exhibited separation. Probability calibration, incremental skill and threshold performance are therefore reported as uncertain and exploratory, not as demonstrated strengths of the framework.

Interpretation. Resampling the available countries may repeat the sole observed incident or omit it; it does not provide additional independent transitions. The reported ROC, calibration, Brier-skill and threshold quantities are retained for transparency only. No probability-performance or deployment claim follows from these results. Reference numbers correspond to the reference list in the main manuscript.
